# Supplementary material for: Comparative Study of Aryl O-, C-, and S-Mannopyranosides as Potential Adhesion Inhibitors toward Uropathogenic E. coli FimH
Source: Molecules. 2019 Oct 2;24(19):3566. doi: 10.3390/molecules24193566 (PMC6804135; doi:10.3390/molecules24193566)
Supplement: Supplementary file 1 [file molecules-24-03566-s001.pdf]

Comparative Study of Aryl *O*-, *C*-, and *S*-Mannopyranosides as Potential Adhesion Inhibitors Toward Uropathogenic *E. coli* FimH

Leila Mousavifar,[a,]* Gérard Vergoten,[c] Guillaume Charron,[a] and René Roy[a,b,d]*

[a] Department of Chemistry, Université du Québec à Montréal, P.O. Box 8888, Succ. Centre-Ville, Montréal, Québec H3C 3P8, Canada. roy.rene@uqam.ca
[b] INRS-Institut Armand-Frappier, Université du Québec, 531 boul. des Prairies, Laval, Québec, H7V 1B7, Canada.
[c] Unité de Glycobiologie Structurale et Fonctionnelle (UGSF), UMR8576 du CNRS, Université de Lille, F-59000 Lille, France;
[d] Glycovax Pharma Inc., 424 Guy, Suite 202, Montreal, Quebec, Canada, H3J 1S6

Supplementary

Contents

General Experimental: S2
1. Synthesis of 6-Aminohexyl α–D-mannopyranoside (4) S4
   Scheme 1 Synthesis of 6-Aminohexyl α–D-mannopyranoside (4) [1–3] S4
   Figure S.1. ^{1}H NMR spectrum of compound S2 ($CDCl_3$, 300 MHz) S5
   Figure S.2 ^{13}C NMR spectrum of compound S2 ($CDCl_3$, 75 MHz) S6
   Figure S.3. ^{1}H NMR spectrum of compound S3 ($CDCl_3$, 300 MHz) S6
   Figure S.4. FTIR spectrum of compound S3 ..... S6
   Figure S.5. ^{13}C NMR spectrum of compound S3 ($CDCl_3$, 75MHz) S6
   Figure S.6. ^{1}H NMR spectrum of compound 4 (CD_3OD, 300 MHz) S7
   Figure S.7. ^{1}HNMR spectrum of compound 4 (CD_3OD, 300 MHz) S8
   Figure S.8. ^{13}C NMR spectrum of compound 4 (CD_3OD, 75 MHz) S8
2 Allyl 2,3,4,6-tetra-O-acetyl-1-thio-α-D-mannopyranoside 9. ..... S8
   Figure S.9. ^{1}H NMR spectrum of compound 9 ($CDCl_3$, 300 MHz) S8
   Figure S10. ^{13}C NMR spectrum of compound 9 ($CDCl_3$, 75 MHz) S9
   Figure S.11. COSY of compound 9 ($CDCl_3$, 300 MHz) S9
   Figure S.12. HSQC of compound 9 ($CDCl_3$, 300 MHz) S10
   Figure S.13. ESI^{+}HRMS spectrum of compound 9 ..... S10
3 (2E)-3-(1,1'-biphenyl-2-propen-1-yl) 2,3,4,6-tetra-O-acetyl-1-thio–α-D-mannopyranoside 11 ..... S10
   Figure S.14. ^{1}H NMR spectrum of compound 11 ($CDCl_3$, 300 MHz) S10

Figure S.15. ^{13}C NMR spectrum of compound 11 ($CDCl_3$, 75 MHz) S11
Figure S.16. ESI$^+$HRMS spectrum of compound 11 .... S11
4 (2*E*)-3-(1,1'-biphenyl-2-propen-1-yl) 1-thio-α-D-mannopyranoside 12. .... S11
Figure S.17. ^{1}H NMR spectrum of compound 12 (CD_3OD, 600 MHz) S11
Figure S.18. ^{13}C NMR spectrum of compound 12 (CD_3OD, 151 MHz) S12
Figure S.19. HPLC-TOF-MAS analysis of fractions of compound 12 .... S12
5 Crystal Data and Structure Refinements S13
Figure S.20. ORTEP diagram for the D mannoside 5.CCDC no: 1840503 .... S13
Thermal ellipsoids are drawn at the 50% probability level. S13
(2*E*)-3-(1, 1'-biphenyl-2-propen-1-yl) α-D-mannopyranoside(5) S14
Figure S.21.ORTEP diagram for the X-Ray structure of *C*-linked mannoside 6.CCDC no: 1871374. Thermal ellipsoids are drawn at the 50% probability level. S15
9-2. (E)-4-[3-(α-D-Mannopyranosyl)prop-1-en-1-yl]-1,1'-biphenyl (6). S16
Figure S.22. Representation of two *O*-linked α–D-mannopyranosides having common 1,1'-biphenyl aglycones; the mannoside residue of compound 5 was superimpose with that of mannoside 7 in the crystalline structure of the protein (PDB 4AV5).[6] S17
Figure S.18 Sensorgram of kinetic analysis of FimH:6-aminohexyl α-D-mannopyranoside affinity by SPR. S17

References

1 S. Tao, T. W. Jia, Y. Yang and L. Q. Chu, *ACS Sensors.*, 2017, **2**, 57–60.

2 V. Dhaware, M. Kar, S. Hotha and S. Sen Gupta, *Langmuir*, 2013, **29**, 5659–5667.

3 K. Öberg, J. Ropponen, J. Kelly, P. Löwenhielm, M. Berglin and M. Malkoch, *Langmuir*, 2013, **29**, 456–465.

4 O. V Dolomanov, L. J. Bourhis, R. J. Gildea, J. A. K. Howard and H. Puschmann, *J. Appl. Cryst.*, 2009, **42**, 339–341.

5 G. M. Sheldrick, *Acta Crystallogr. Sect. A*, 2015, **71**, 3–8.

6 A. Wellens, M. Lahmann, M. Touaibia, J. Vaucher, S. Oscarson, R. Roy, H. Remaut and J. Bouckaert, *Biochemistry*, 2012, **51**, 4790–4799.

General Experimental:

Reactions were carried out under Nitrogen using commercially available ACS grade solvents which were stored over 4 Ǻ molecular sieves. Solutions in organic solvents were dried over anhydrous Na_2SO_4, filtered, and concentrated under reduced pressure. Reagents were obtained from Sigma

Aldrich. Reactions were monitored by thin-layer chromatography using silica gel 60 F254 coated plates (E. Merck). NMR spectra were recorded on Varian Inova AS600 and Bruker Avance III HD 600 MHz spectrometer. Proton and carbon chemical shifts (δ) are reported in ppm relative to the chemical shift of residual $CHCl_3$, which was set at 7.28 ppm (^{1}H) and 77.16 ppm (^{13}C{H}). Coupling constants (J) are reported in Hertz (Hz) and the following abbreviations are used for peak multiplicities: singlet (s), doublet (d), doublet of doublets (dd), doublet of doublet with equal coupling constants (t_{ap}), triplet (t), multiplet (m). Assignments were made using COSY (Correlated SpectroscopY) and HSQC (Heteronuclear Single Quantum Coherence) experiments. High-resolution mass spectra (HRMS) were measured with a LC-MS-TOF (Liquid Chromatography Mass Spectrometry Time of Flight) instrument (Agilent Technologies) in positive electrospray mode by the analytical platform of UQAM. SPR were performed with a Biacore T200 on a CM5 sensor chip (GE Healthcare Life Sciences). Optical rotations were measured with a JASCO P-1010 polarimeter. Melting points were measured on a Fisher Jones apparatus.

A. General procedure for de-*O*-acetylation.

The acetylated mannosides **S4 and 11** were dissolved in dry MeOH (3 mL), a solution of sodium methoxide (1 M in MeOH, 0.5 equiv) was added and the reaction mixture was stirred at room temperature until disappearance of the starting material. The solution was neutralized by addition of ion-exchange resin (Amberlite IR 120), filtered, washed with MeOH and then the solvent was removed in vacuum compounds **4** and **12**. Lyophilization of **4** yielded the fully deprotected mannoside desired compound. Compound **12** was purified by reversed phase high performance liquid chromatography (RP-HPLC) (A: H_2O + 0.1% trifluoroacetic acid, B: ACN + 0.1% trifluoroacetic acid, 5 mL/min).

1. **Synthesis of 6-Aminohexyl α–D-mannopyranoside (4)**

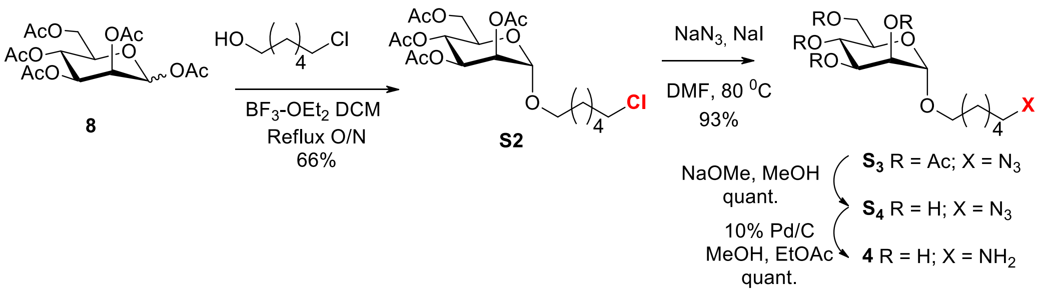

Scheme 1 Synthesis of 6-Aminohexyl α–D-mannopyranoside (4) [1–3]

6-Chlorohexyl 2,3,4,6-tetra-O-acetyl-α-D-mannopyranoside (S2)

Penta-*O*-acetyl-mannopyranose (**8**) (507 mg, 1.30 mmol, 1.0 eq.) and 6-chlorohexanol (340 µL, 2.56 mmol, 2. 0 eq.) were dissolved in dry DCM (10 mL) at 0 °C. $Et_2O.BF_3$ (480 µL, 3.90 mmol, 3.0 eq.) was added dropwise. The mixture was stirred at 0 °C for 1 h and then at 40 °C for 17 h. The solution was neutralized by adding a saturated solution of $NaHCO_3$ (10 mL). The organic phase was separated, washed with water and dried over sodium sulfate. The DCM was removed under vacuum. The crude residue was purified by silica gel column chromatography (Petroleum ether: EtOAc, 3:2) to afford the pure title compound as a yellow oil (404 mg, 0.895 mmol) in 66 % yield; R_f = 0.62 (Petroleum ether: EtOAc, 3:2). ^{1}H NMR (300 MHz, $CDCl_3$): δ 5.36 (dd, 1H, $J_{2,3}$= 3 Hz, $J_{3,4}$ = 9 Hz, H-3), 5.26- 5.21 (m, 2H, H-4, H-2), 4.79 (d, 1H, $J_{1,2}$ = 1.5 Hz, H-1), 4.27 (dd, 1H, $J_{6a, 6b}$ = 12.2 Hz, $J_{5, 6a}$ = 5.3 Hz, H-6a), 4.09 (dd, 1H, $J_{6a, 6b}$ = 12.2 Hz, $J_{5, 6b}$= 2.3 Hz, H-6b), 3.95 (ddd, 1H, $J_{4, 5}$ = 8.9 Hz, $J_{5, 6a}$ = 5.4 Hz, $J_{5, 6b}$ = 2.4 Hz, H-5), 3.72-3.64 (m, 1H. OC*H*H), 3.53 (t, 1H, J_{H-H} = 6Hz, -CH_2Cl), 3.44 (m, 1H, OCH*H*), 2.14 (s, 3H, OAc), 2.09 (s, 3H, OAc), 2.03 (s, 3H, OAc), 1,98 (s, 3H, OAc), 1.80-1.73 (m, 2H), 1.72 - 1.59 (m, 2H), 1.42 - 1.40 (m, 4H). ^{13}C{^{1}H} NMR ($CDCl_3$,): δ 170.6-169.7 (4C=O), 97.6; 69.7; 69.1; 68.4 (CH_2-O); 68.3, 66.3, 62.5; 45.0, 32.4 (CH_2), 29.1 (CH_2), 26.6 (CH_2), 25.4 (CH_2), 20.9-20.7 (4 OCOCH_3). Other physical data matched those of the literature.[2]

6-Azidohexyl 2, 3, 4, 6-tetra-O-acetyl-α-D-mannopyranoside (S3).

The above 6-chlorohexyl derivative S2 (551 mg, 1.18 mmol, 1.0 eq.) was dissolved in dry DMF (5 mL). Sodium azide (385 mg, 5.92 mmol, 5.0 eq.) and sodium iodide (40 mg, 0.22 eq.) were added to the mixture. The solution was stirred at 80 °C for 24 h and then at room temperature for 16 h. The mixture was diluted with water and the aqueous phase was extracted 3 times with EtOAc. The organic phase was washed with water, dried and concentrated under reduced pressure. The product was purified by silica gel column chromatography (Toluene, EtOAc, 3:2) to yield pure title compound as a yellow oil (521 mg, 1.10 mmol, 93 %); R_f = 0.67 (Toluene, EtOAc, 3:2). 521 mg, 1.10 mmol, IR: (2104 cm^{-1} for N_3). ^{1}H NMR (300 MHz,

$CDCl_3$): δ 5.36 (dd, 1H, $J_{2,3}$= 3.3 Hz, $J_{3,4}$ = 10 Hz, H-3), 5.32-5.22 (m, 2H, H-4, H-2), 4.81(d, 1H, $J_{1,2}$ = 1.6 Hz, H-1), 4.29 (dd, 1H, $J_{6a, 6b =}$ 12.2 Hz $J_{5, 6a}$ = 5.3 Hz, H-6a), 4.09 (dd, 1H, $J_{6a, 6b}$ = 12.2 Hz, $J_{5, 6b}$ = 2.4 Hz, H-6b), 3.98 (ddd, 1H, $J_{4, 5}$ = 9.4 Hz, $J_{5, 6a}$ = 5.3 Hz, $J_{5, 6b}$ = 2.4 Hz, H-5), 3.76-3.66 (m, 1H. OC*H*H), 3.49-3.42 (m, 1H, OCH*H*), 3.29 (t, 1H, $J_{H\text{-}H}$ = 6.8 Hz, -CH_2-N_3), 2.15 - 1,99 (s, 12H, OAc. 1.73 – 1.50 (m, 4H), 1.50 - 1.29 (m, 4H). $^{13}C\{^1H\}$ NMR ($CDCl_{3,}$): 170.6 - 169.7 (4×CO), 97.6 (CH_1), 69.7; 69,1, 68.4, 68.3, 66.3, 62.5, 51.3 (CH_2-N_3), 29.1 (CH_2), 28.7 (CH_2), 26.5 (CH_2), 25.7 (CH_2), 20. 9- 20.7 (4× OCO*C*H̲3).[2]

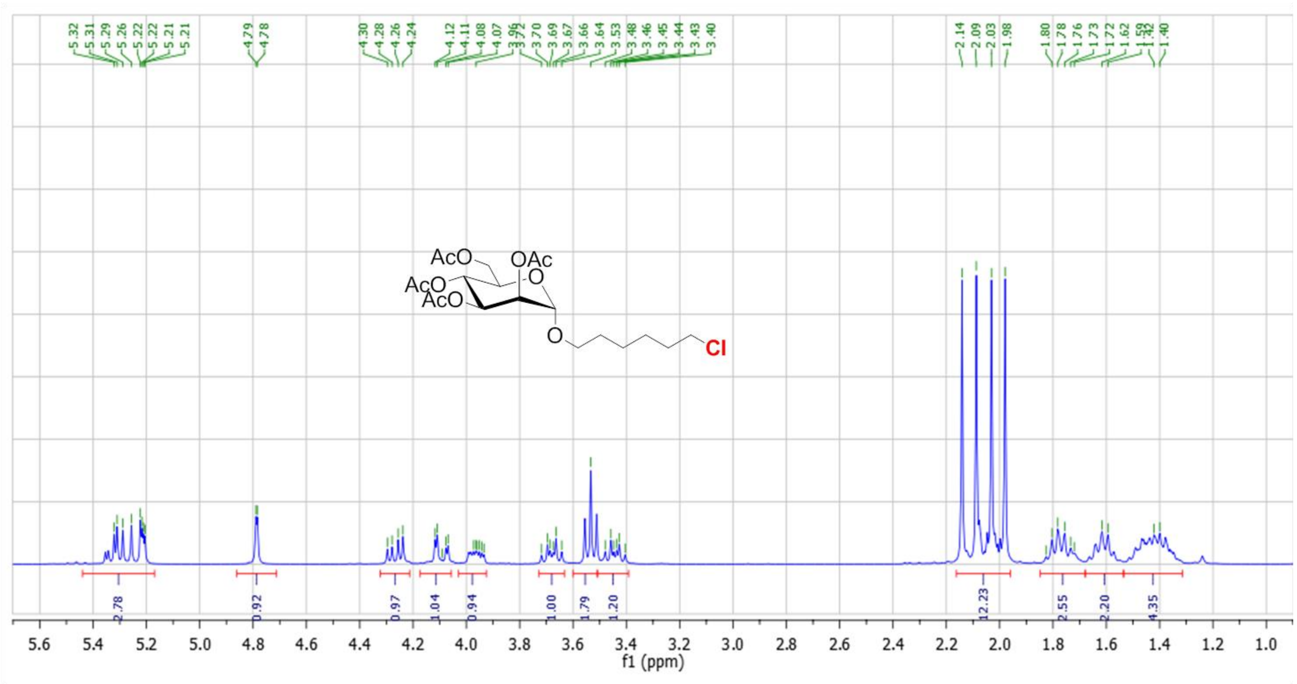

Figure S.1. ^{1}H NMR spectrum of compound S2 ($CDCl_3$, 300 MHz)

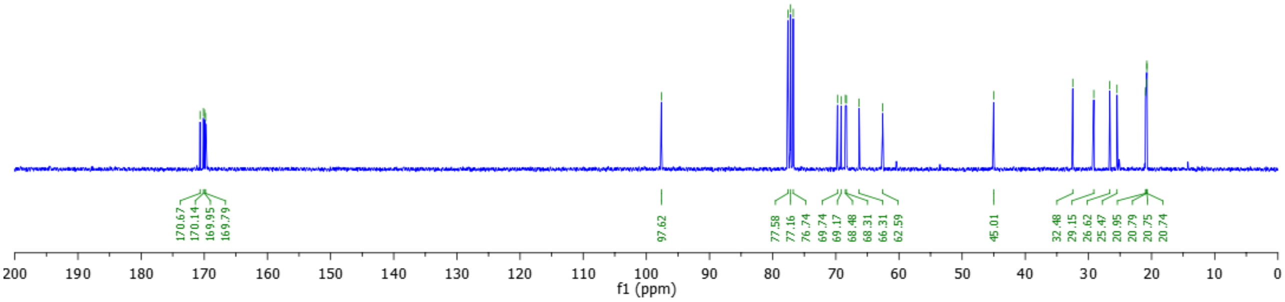

Figure S.2 ^{13}C NMR spectrum of compound S2 ($CDCl_3$, 75 MHz)

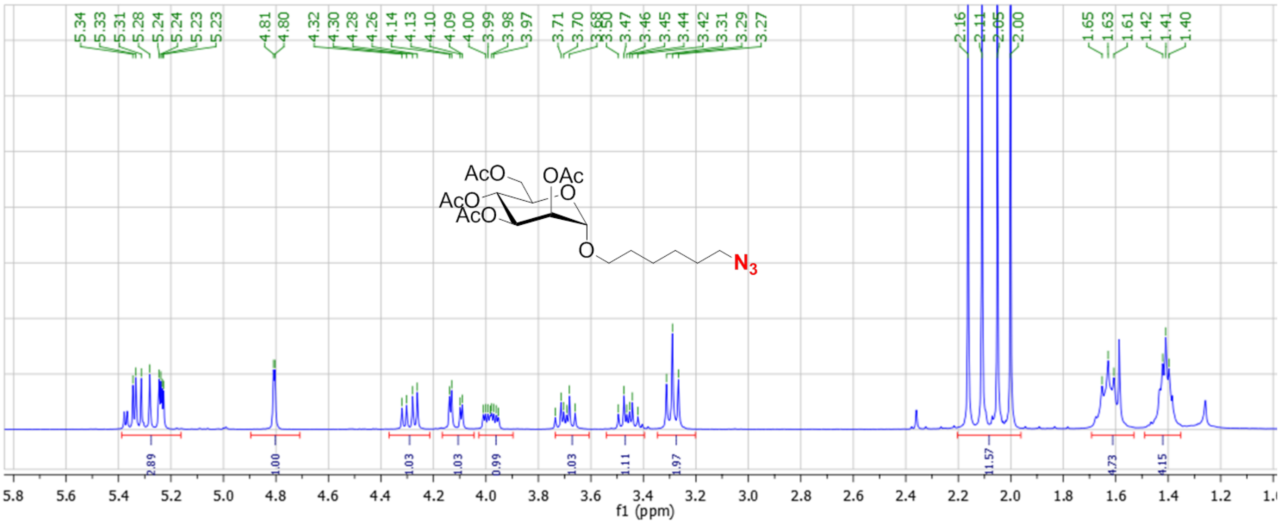

Figure S.3. ^{1}H NMR spectrum of compound S3 ($CDCl_3$, 300 MHz)

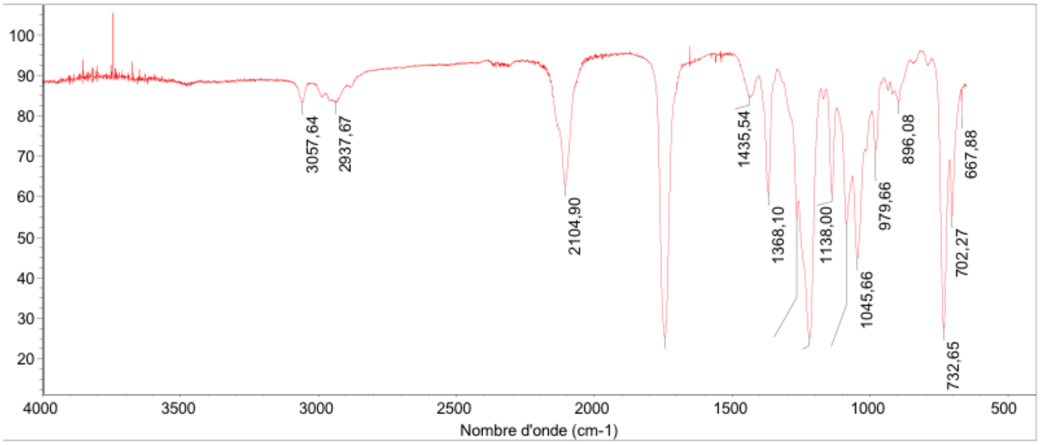

Figure S.4. FTIR spectrum of compound S3

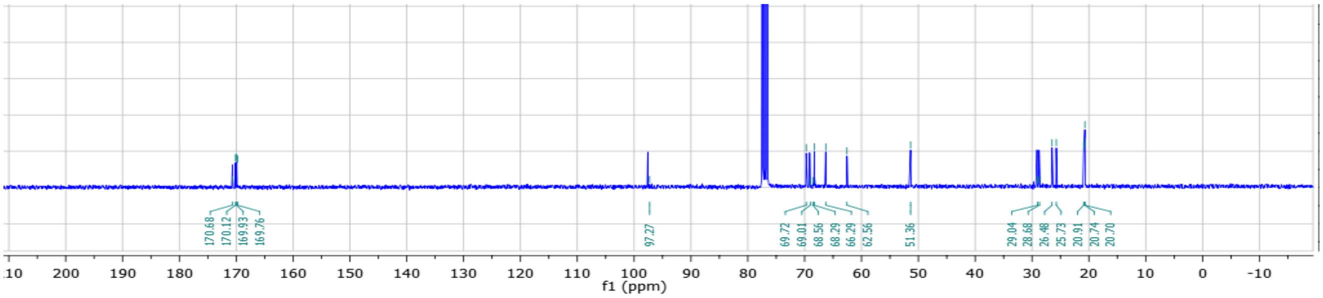

Figure S.5. ^{13} C NMR spectrum of compound S3 ($CDCl_3$, 75MHz)

6-Azidohexyl α-D-mannopyranoside (4).

This compound was prepared according to the published procedure.[3] ^{1}H NMR (300 MHz, CD_3OD) δ: 4.75 (d, 1H), 3.82-3.63 (m, 8H), 3. 31 (t, J = 6.9 Hz, 2H), 1.69 – 1.48 (m, 4H), 1.45 – 1.29 (m, 4H). Other physical data matched those of the literature.

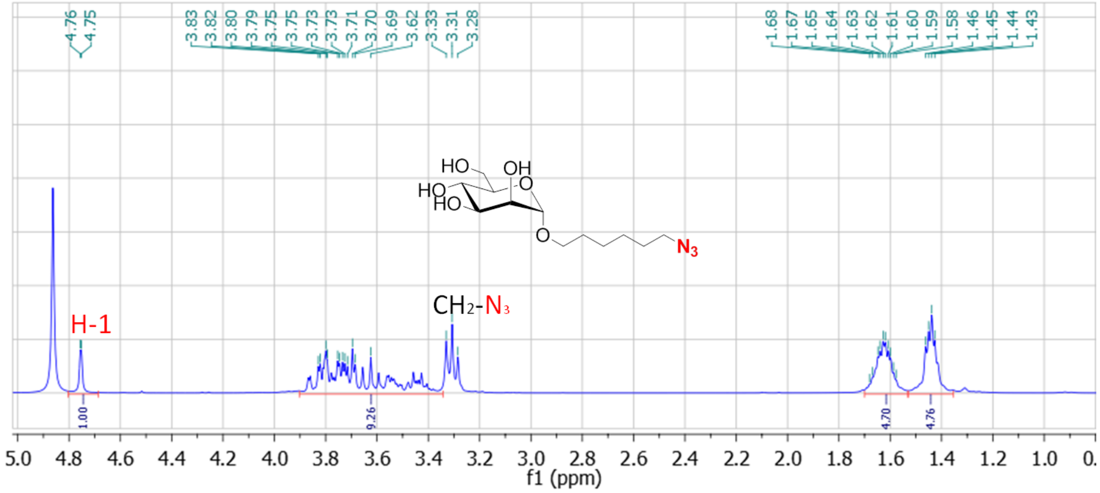

Figure S.6. ^{1}H NMR spectrum of compound 4 (CD_3OD, 300 MHz)

6-Aminohexyl α-D-mannopyranoside (4).

This compound was prepared according to the published procedure.[3] ^{1}H NMR (300 MHz, CD_3OD) δ: 4.72 (d, 1H), 3.92-3.81 (m, 2H), 3.79-3.65 (m, 3H), 3.65-3.56 (m, 2H), 3.56 – 3.47 (m, 1H), 2.62 (t, J = 7.9 Hz, 2H), 1.61-1.37 (m, 10H). $^{13}C\{^1H\}$ NMR (CD_3OD): δ 100.15, 73.2, 71.2, 70.8, 67.2, 67.0, 61.5, 40.8, 31.4, 29.0, 29.0, 26.2, 25.7.

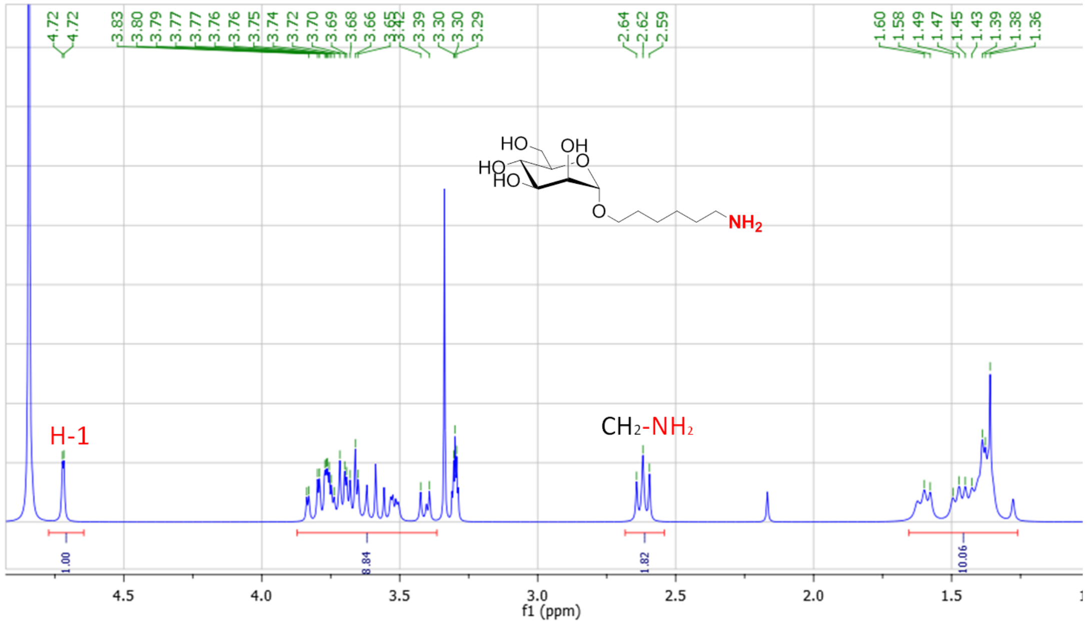

Figure S.7. ^{1}HNMR spectrum of compound 4 (CD_3OD, 300 MHz)

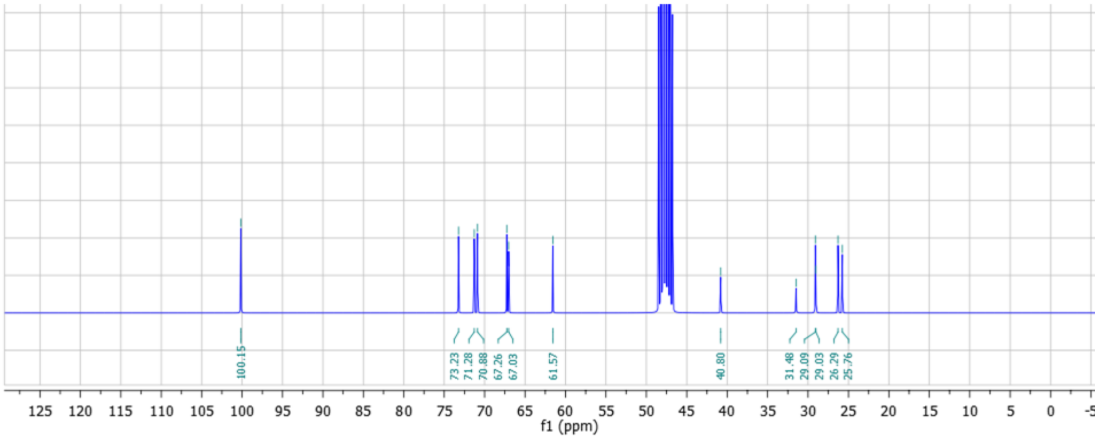

Figure S.8. ^{13}C NMR spectrum of compound 4 (CD_3OD, 75 MHz)

2 Allyl 2,3,4,6-tetra-O-acetyl-1-thio-α-D-mannopyranoside 9.

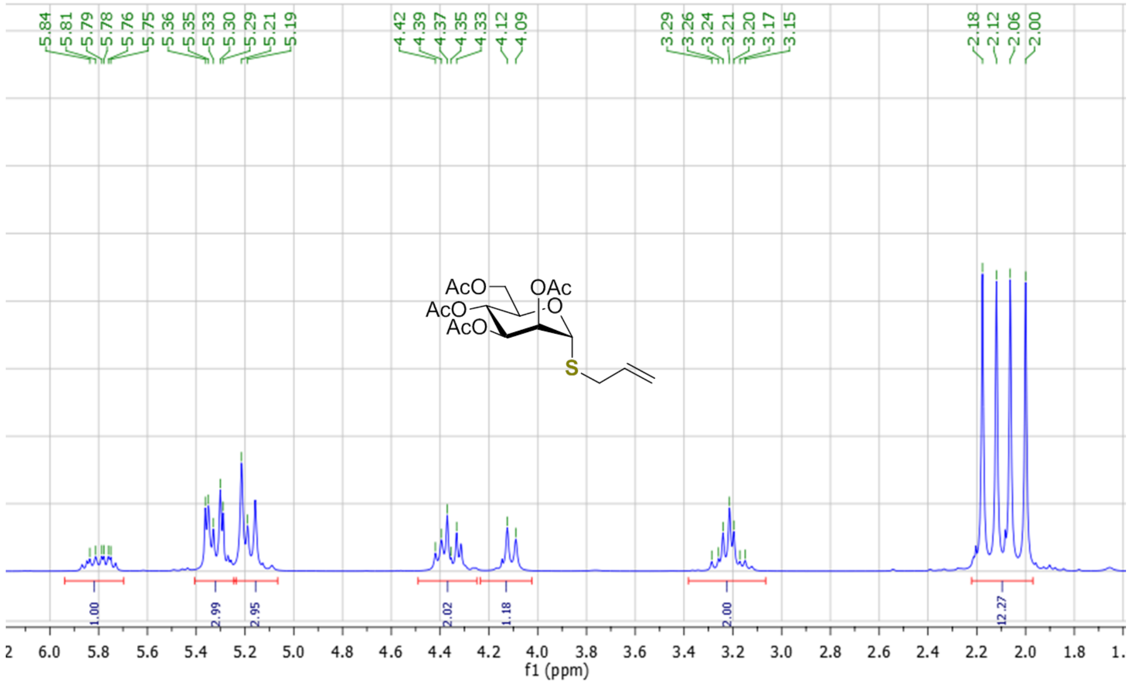

Figure S.9. ^{1}H NMR spectrum of compound 9 ($CDCl_3$, 300 MHz)

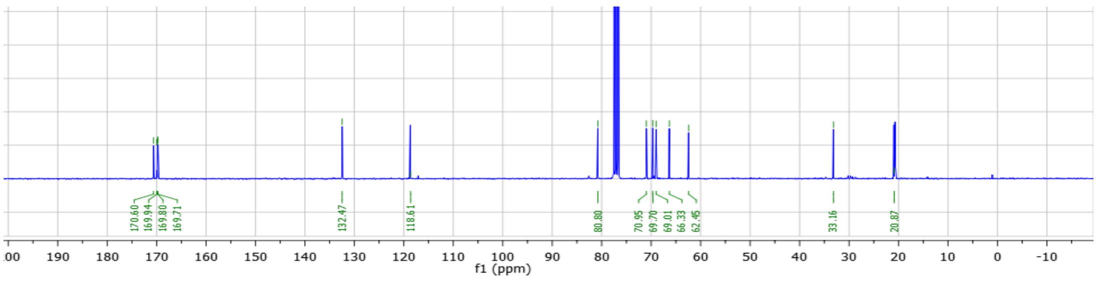

Figure S10. **^{13}C NMR spectrum of compound 9 ($CDCl_3$, 75 MHz)**

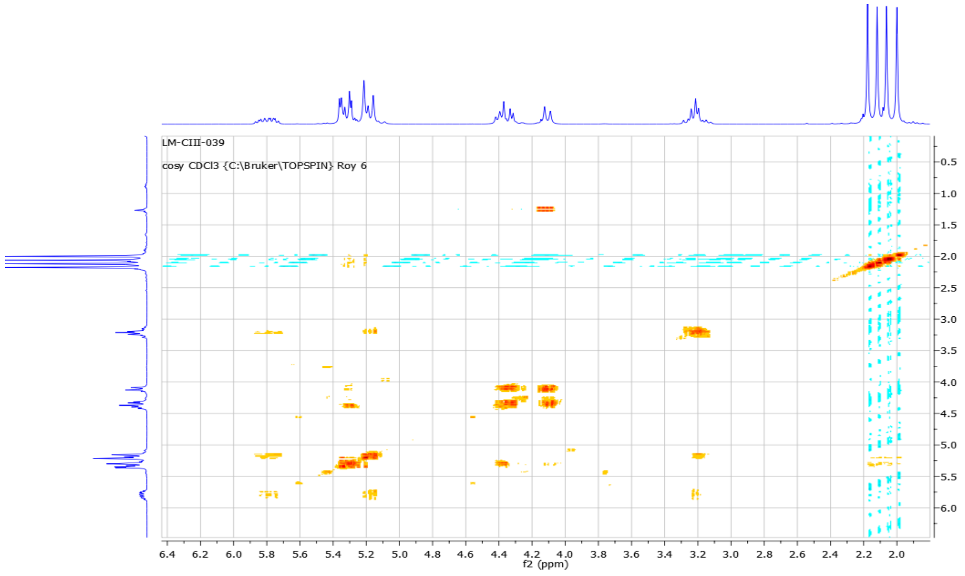

Figure S.11. COSY of compound 9 ($CDCl_3$, 300 MHz)

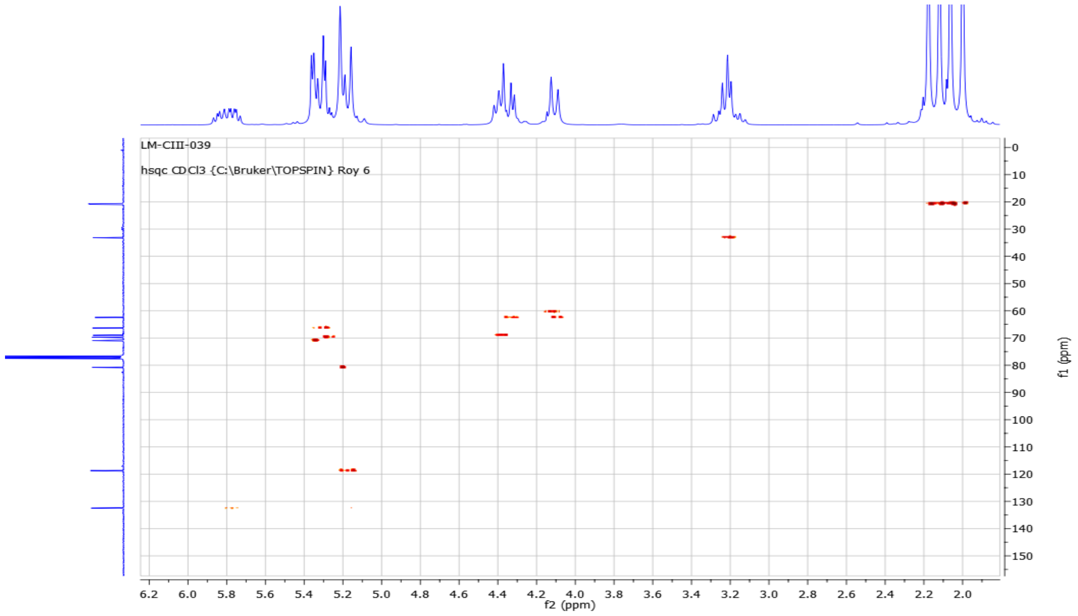

Figure S.12. HSQC of compound 9 ($CDCl_3$, 300 MHz)

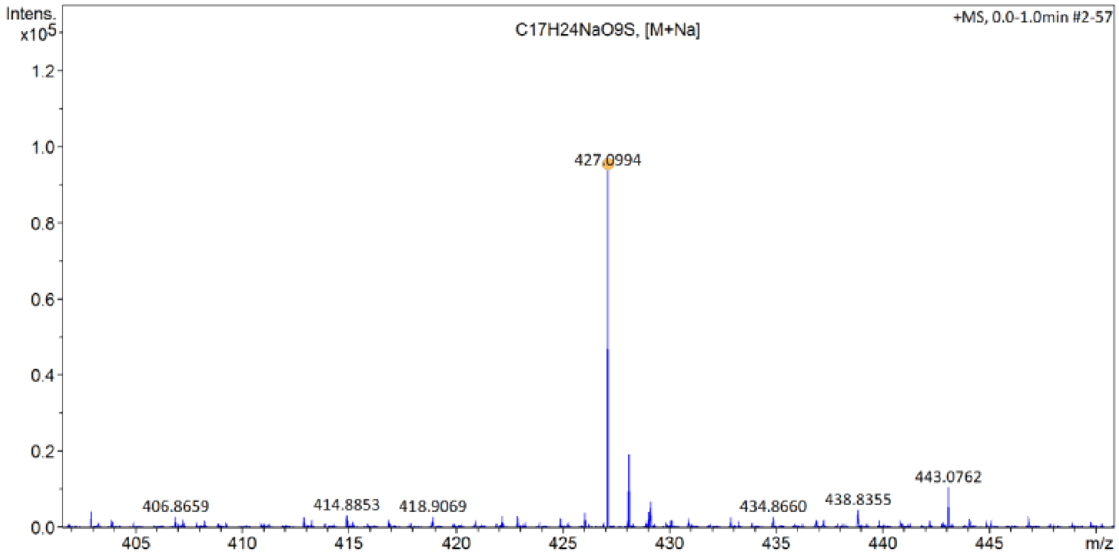

Figure S.13. ESI$^+$HRMS spectrum of compound 9

3 (2E)-3-(1,1'-biphenyl-2-propen-1-yl) 2,3,4,6-tetra-O-acetyl-1-thio–α-D-mannopyranoside 11

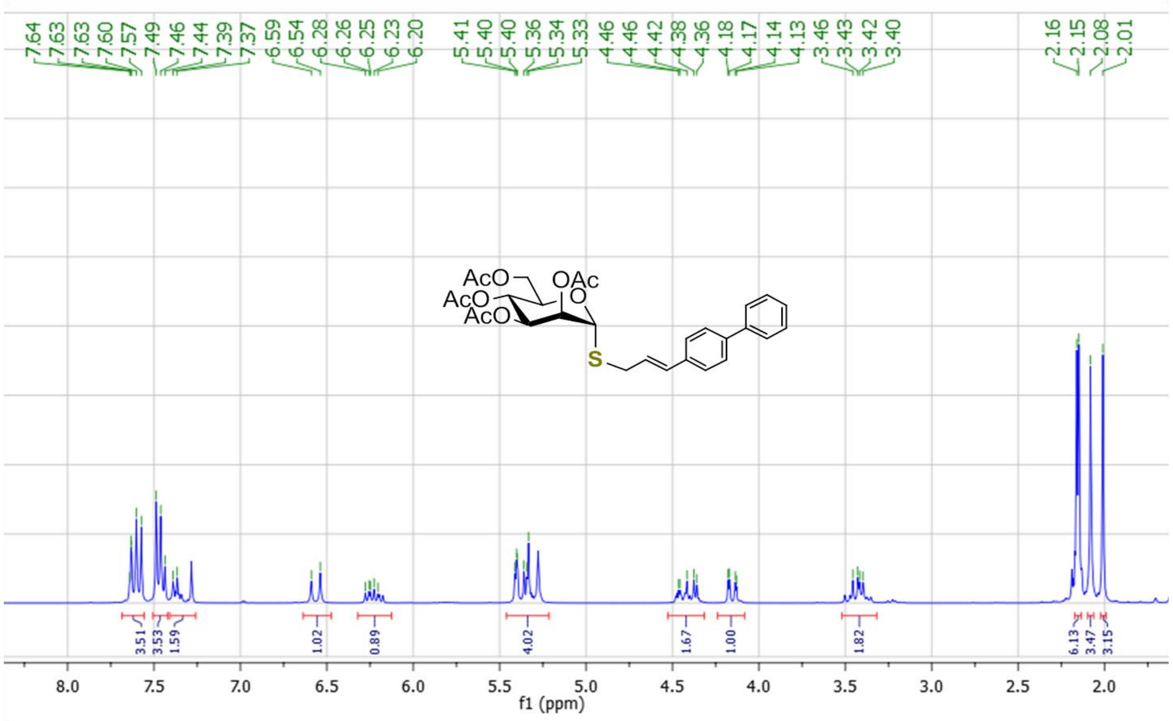

Figure S.14. ^{1}H NMR spectrum of compound 11 ($CDCl_3$, 300 MHz)

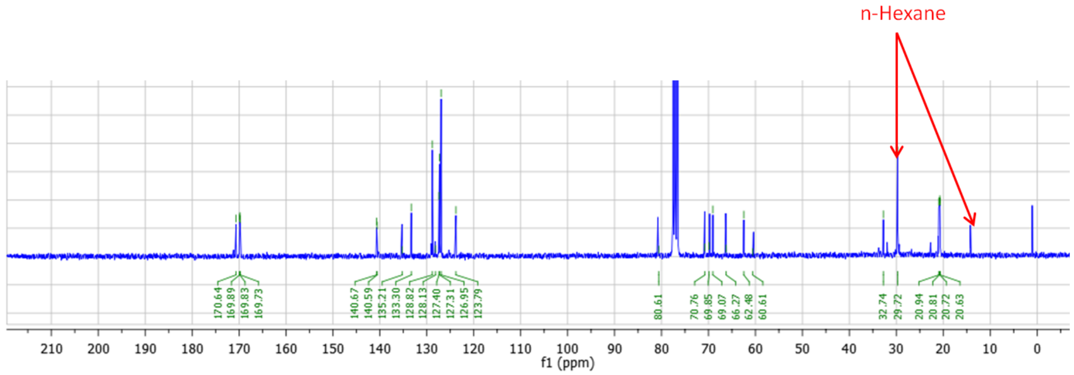

Figure S.15. ^{13}**C NMR spectrum of compound 11 ($CDCl_3$, 75 MHz)**

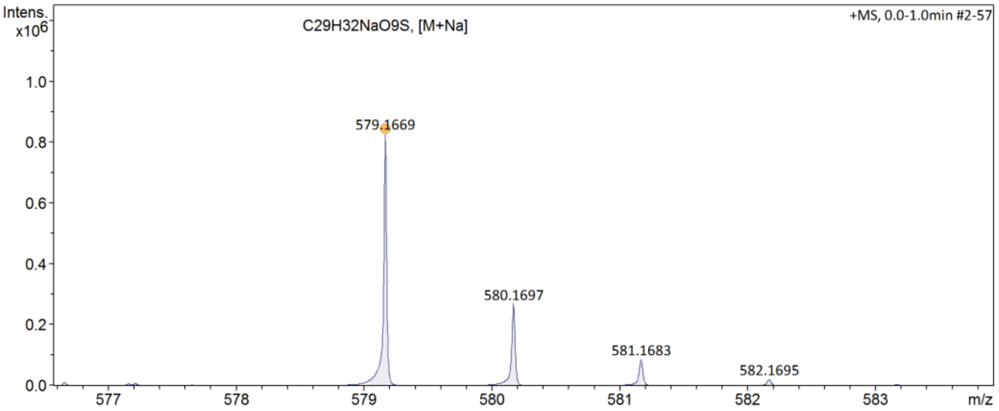

Figure S.16. ESI$^+$HRMS spectrum of compound 11

4 (2*E*)-3-(1,1'-biphenyl-2-propen-1-yl) 1-thio-α-D-mannopyranoside 12.

This compound was deprotected according to general procedure **B**.

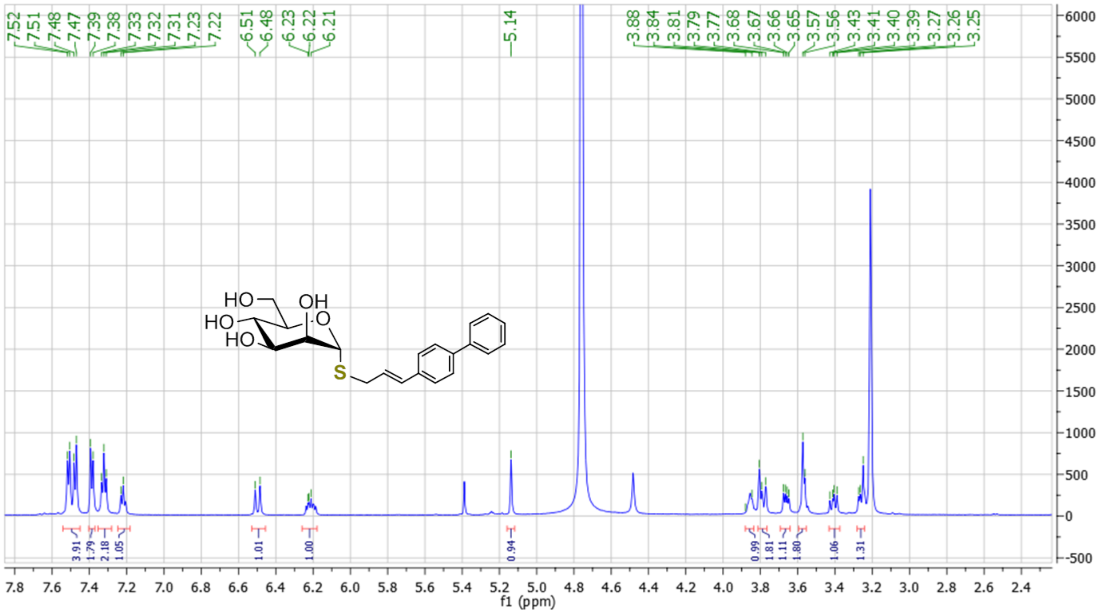

Figure S.17. ^{1}**H NMR spectrum of compound 12 (CD_3OD, 600 MHz)**

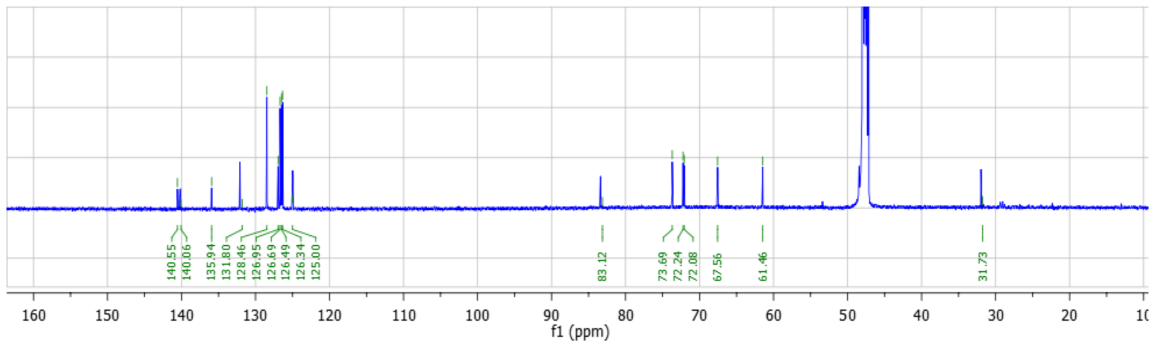

Figure S.18. ^{13}**C NMR spectrum of compound 12 (CD**$_3$**OD, 151 MHz)**

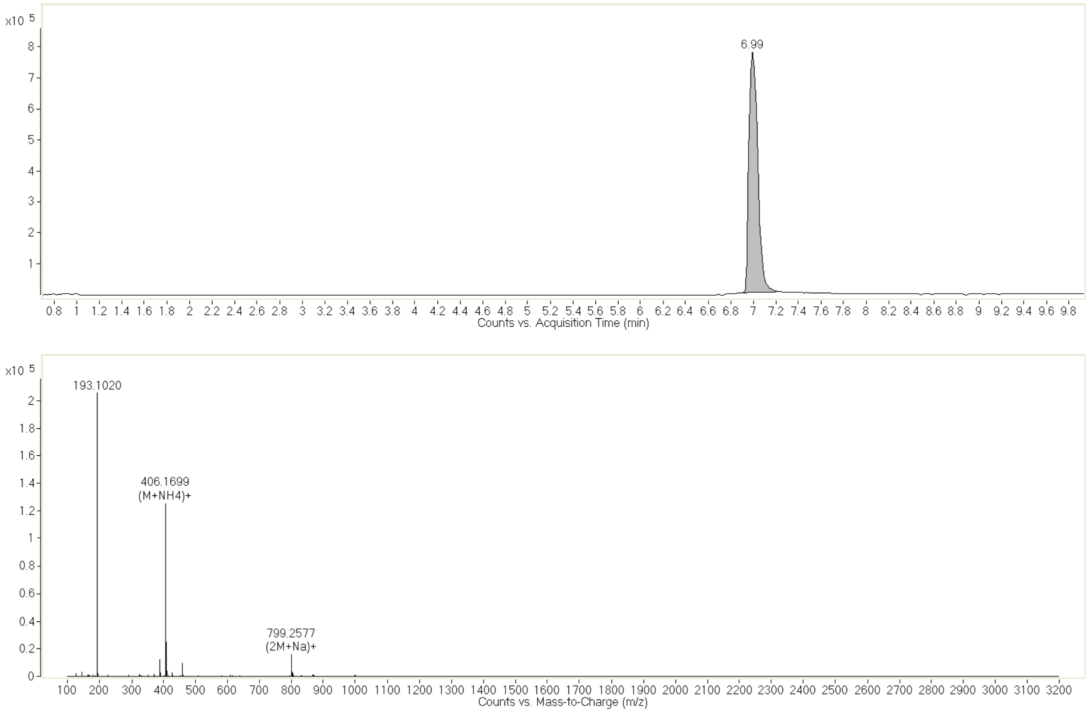

Figure S.19. HPLC-TOF-MAS analysis of fractions of compound 12

5 Crystal Data and Structure Refinements

(2*E*)-3-(1, 1'-biphenyl-2-propen-1-yl) α-D-mannopyranoside

Single crystals of $C_{21}H_{24}O_6$ were crystallized from DCM/MeOH. A suitable crystal was selected and mounted on a cryoloop on a Bruker Venture Metal jet diffractometer. The crystal was kept at 150 K during data collection. Using Olex2 [4], the structure was solved with the XT [5] structure solution program using Intrinsic Phasing and refined with the XL [5] refinement package using Least Squares minimization.

Crystal Data for $C_{21}H_{24}O_6$ (M =372.40 g/mol): orthorhombic, space group $P2_12_12_1$ (no. 19), a = 6.2463(3) Å, b = 7.5145(3) Å, c = 39.1736(18) Å, V = 1838.72(14) Å^3, Z = 4, T = 150 K, μ(GaKα) = 0.520 mm^{-1}, *Dcalc* = 1.345 g/cm^3, 22152 reflections measured (3.924° ≤ 2Θ ≤ 107.97°), 3372 unique (R_{int} = 0.0551, R_{sigma} = 0.0354) which were used in all calculations. The final R_1 was 0.0494 (I > 2σ(I)) and wR_2 was 0.1208 (all data).

Crystallographic data for the structure reported in this paper has been deposited at the Cambridge Crystallographic Data Centre (CCDC) with deposition no: 1840503 for $C_{21}H_{24}O_6$. Supplementary data can be obtained free of charge from CCDC, 12 Union Road, Cambridge CB2 1EZ, UK (fax: (+44)1223-336-033; e-mail: deposit@ccdc.cam.ac.uk

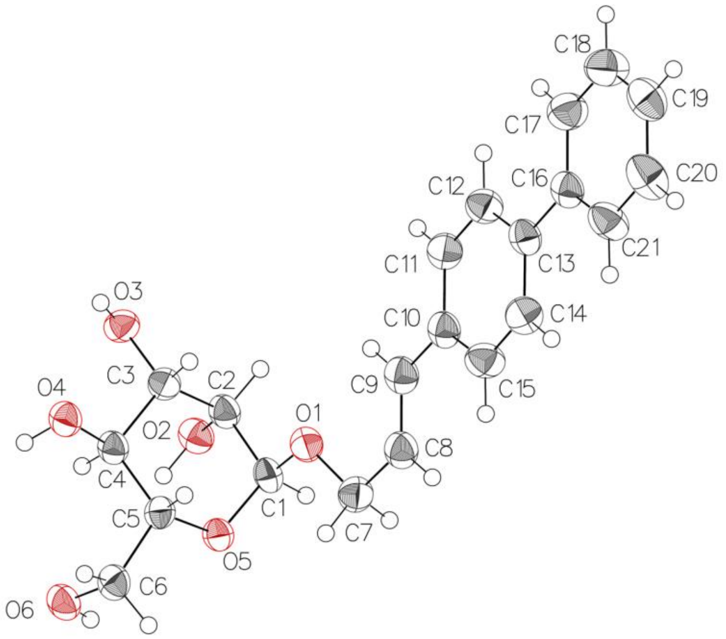

Figure S.20. ORTEP diagram for the D mannoside 5.CCDC no: 1840503 Thermal ellipsoids are drawn at the 50% probability level.

(2*E*)-3-(1, 1’-biphenyl-2-propen-1-yl) α-D-mannopyranoside(5)

Bond precision: C-C = 0.0056 A Wavelength=1.34139

Cell:	a=6.2463(3)	b=7.5145(3)	c=39.1736(18)
	alpha=90	beta=90	gamma=90
Temperature:	150 K		

	Calculated	Reported
Volume	1838.72(14)	1838.72(14)
Space group	P 21 21 21	P 21 21 21
Hall group	P 2ac 2ab	P 2ac 2ab
Moiety formula	C21 H24 O6	C21 H24 O6
Sum formula	C21 H24 O6	C21 H24 O6
Mr	372.40	372.40
Dx,g cm-3	1.345	1.345
Z	4	4
Mu (mm-1)	0.516	0.520
F000	792.0	792.0
F000’	794.00	
h,k,lmax	7,9,47	7,9,47
Nref	3376[2007]	3372
Tmin,Tmax	0.954,0.985	0.489,0.751
Tmin’	0.834	

Correction method= # Reported T Limits: Tmin=0.489 Tmax=0.751
AbsCorr = MULTI-SCAN

Data completeness= 1.68/1.00 Theta(max)= 53.985

R(reflections)= 0.0494(2868) wR2(reflections)= 0.1228(3372)

S = 1.048 Npar= 261

The following ALERTS were generated. Each ALERT has the format
test-name_ALERT_alert-type_alert-level.
Click on the hyperlinks for more details of the test.

(E)-4-[3-(α-D-Mannopyranosyl)prop-1-en-1-yl]-1,1'-biphenyl (6).

Single crystals of $C_{21}H_{24}O_5$ were crystallized from MeOH. A suitable crystal was selected and mounted on a cryoloop on a Bruker Venture Metaljet diffractometer. The crystal was kept at 150 K during data collection. Using Olex2 [4], the structure was solved with the XT [5] structure solution program using Intrinsic Phasing and refined with the XL [5] refinement package using Least Squares minimization.

Crystal Data for $C_{21}H_{24}O_5$ (M =356.40 g/mol): monoclinic, space group P21 (no. 4), a = 9.7448(4) Å, b = 8.1887(4) Å, c = 21.6060(9) Å, β = 92.097(2)°, V = 1722.94(13) Å3, Z = 4, T = 150 K, μ(GaKα) = 0.506

mm-1, Dcalc = 1.374 g/cm3, 46514 reflections measured (3.56° ≤ 2Θ ≤ 121.384°), 7923 unique (Rint = 0.0693, R sigma = 0.0486) which were used in all calculations. The final R1 was 0.0518 (I > 2σ(I)) and wR2 was 0.1405.

Crystallographic data for the structure reported in this paper has been deposited at the Cambridge Crystallographic Data Centre (CCDC) with deposition no: 1871374 for C21H24O5. Supplementary data can be obtained free of charge from CCDC, 12 Union Road, Cambridge CB2 1EZ, UK (fax: (+44)1223-336-033; e-mail: deposit@ccdc.cam.ac.uk

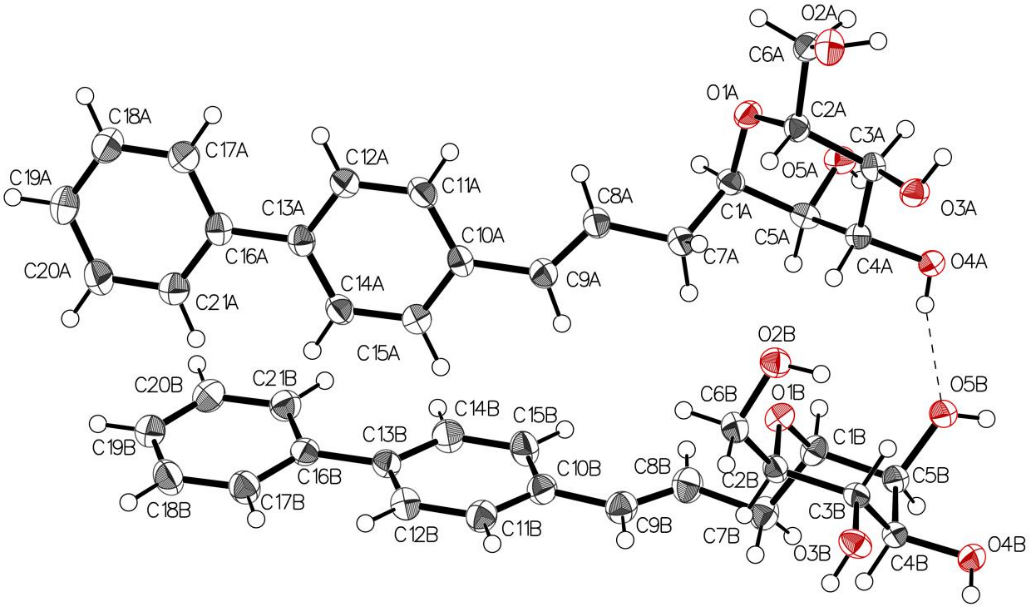

Figure S.21.ORTEP diagram for the X-Ray structure of *C*-linked mannoside 6.CCDC no: 1871374. Thermal ellipsoids are drawn at the 50% probability level.

5-1. (E)-4-[3-(α-D-Mannopyranosyl)prop-1-en-1-yl]-1,1'-biphenyl (6).

```
Bond precision:   C-C = 0.0053 A          Wavelength=1.34139

Cell:             a=9.7448(4)      b=8.1887(4)        c=21.6060(9)
                  alpha=90         beta=92.097(2)     gamma=90
Temperature:      150 K

                Calculated                  Reported
Volume          1722.95(13)                 1722.94(13)
Space group     P 21                        P 1 21 1
Hall group      P 2yb                       P 2yb
Moiety formula  C21 H24 O5                  C21 H24 O5
Sum formula     C21 H24 O5                  C21 H24 O5
Mr              356.40                      356.40
Dx,g cm-3       1.374                       1.374
Z               4                           4
Mu (mm-1)       0.506                       0.506
F000            760.0                       760.0
F000'           761.85
h,k,lmax        12,10,28                    12,10,28
Nref            7934[ 4247]                 7923
Tmin,Tmax       0.947,0.955                 0.416,0.516
Tmin'           0.936

Correction method= # Reported T Limits: Tmin=0.416 Tmax=0.516
AbsCorr = MULTI-SCAN

Data completeness= 1.87/1.00        Theta(max)= 60.692

R(reflections)= 0.0518( 6500)       wR2(reflections)= 0.1405( 7923)

S = 0.913                  Npar= 503
```

The following ALERTS were generated. Each ALERT has the format
test-name_ALERT_alert-type_alert-level.
Click on the hyperlinks for more details of the test.

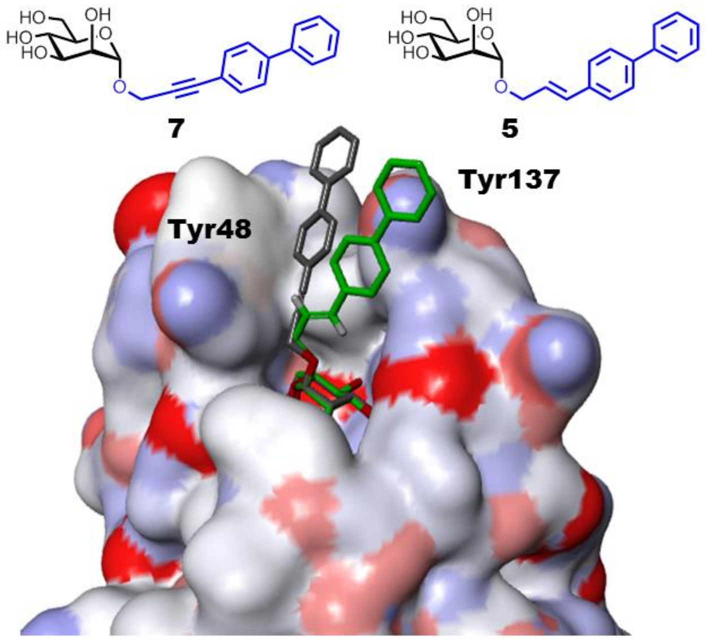

Figure S.22. Representation of two *O*-linked α–D-mannopyranosides having common 1,1'-biphenyl aglycones; the mannoside residue of compound 5 was superimpose with that of mannoside 7 in the crystalline structure of the protein (PDB 4AV5).[6]

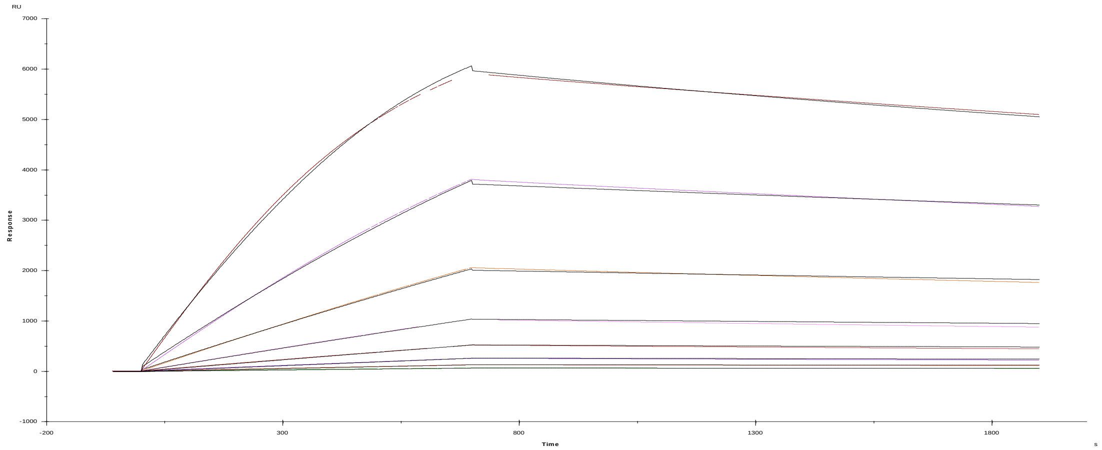

Residuals:

Fitted constants:

ka (1/Ms)	Kd (1/s)	KD (M)	Rmax (RU)	tc	Chi² (RU²)	U-value
5343	2,06E-04	3,86E-08	7129	5,75E+06	783	1

Figure S.23. Sensorgram of kinetic analysis of FimH:6-aminohexyl α-D-mannopyranoside affinity by SPR.
